# The Performance of Pleural Fluid T-SPOT.TB Assay for Diagnosing Tuberculous Pleurisy in China: A Two-Center Prospective Cohort Study

**DOI:** 10.3389/fcimb.2019.00010

**Published:** 2019-01-30

**Authors:** Ying Luo, Yaoju Tan, Jing Yu, Qun Lin, Hongyan Hou, Liyan Mao, Weiyong Liu, Feng Wang, Ziyong Sun

**Affiliations:** ^1^Department of Laboratory Medicine, Tongji Hospital, Tongji Medical College, Huazhong University of Science and Technology, Wuhan, China; ^2^Department of Clinical Laboratory, Guangzhou Chest Hospital, Guangzhou, China

**Keywords:** PB T-SPOT, PF T-SPOT, IFN-γ, pleural tuberculosis, diagnosis

## Abstract

The performance of T-SPOT.TB (T-SPOT) assay in diagnosing pleural tuberculosis (plTB) is inconsistent. In this study, we compared the performance of peripheral blood (PB) and pleural fluid (PF) T-SPOT assay in diagnosing plTB. Between July 2017 and March 2018, 218 and 210 suspected plTB patients were prospectively enrolled from Wuhan (training) and Guangzhou (validation) cohort, respectively. PB T-SPOT, PF T-SPOT, and other conventional tests were simultaneously performed. Our data showed the performance of PB T-SPOT in diagnosing plTB was limited, especially with low sensitivity. However, the results of early secreted antigenic target 6 (ESAT-6) and culture filtrate protein 10 (CFP-10) in PF T-SPOT were significantly increased compared with those in PB T-SPOT in plTB patients. If using 76 as the cutoff value of MAX (the larger of ESAT-6 and CFP-10) in Wuhan cohort, the sensitivity and specificity of PF T-SPOT to diagnose plTB were 89.76 and 96.70%, respectively. The diagnostic accuracy of PF T-SPOT was better than other routine tests such as pathogen detection methods and biochemical markers. The diagnostic accuracy of PF T-SPOT in Guangzhou cohort was similar to that in Wuhan cohort, with a sensitivity and specificity of 91.07 and 94.90%, respectively. Furthermore, CD4^+^ T cells were more activated in PF compared with PB, and the frequency of *mycobacterium tuberculosis*-specific CD4^+^ T cells in PF was significantly higher than that in PB in plTB patients. In conclusion, the performance of PF T-SPOT is obviously better than PB T-SPOT or other laboratory tests, which suggests that PF T-SPOT assay has been of great value in the diagnosis of pleural tuberculosis.

## Introduction

Tuberculosis (TB) is an infectious disease caused by *Mycobacterium tuberculosis* (Mtb). It remains a major cause of morbidity and mortality worldwide (World Health Organization, 2017). During the last 20 years, extrapulmonary TB has become more and more common in most countries (Kruijshaar and Abubakar, 2009; Sandgren et al., 2013). However, pleural TB (plTB), as one of the most common forms of extrapulmonary TB (Valdes et al., 1998; Peto et al., 2009; Porcel, 2009; Light, 2010), is still difficult to diagnose.

Prompt diagnosis is important for optimal treatment in plTB patients. Unfortunately, the currently available tests are unsatisfactory (Norbis et al., 2013). The sensitivity of acid-fast staining (AFS) and Mtb culture is low, which is may be due to the pauci-bacillary nature of the disease (Dunlap et al., 2000; Ruan et al., 2012; Vorster et al., 2015). The Xpert MTB/RIF (Xpert) assay is highly recommended for rapid diagnosis of pulmonary TB (Ho et al., 2016; Pan et al., 2018), while World Health Organization acknowledged the low quality of evidence supporting the use of this assay to diagnose plTB (Tortoli et al., 2012; World Health Organization, 2014b). Some other commonly used indexes, such as percentage of lymphocyte and adenosine deaminase (ADA) level in pleural effusion, have limited value in diagnosing plTB because of low sensitivity or specificity (Greco et al., 2003; World Health Organization, 2014a).

T-SPOT.TB (T-SPOT) assay, which uses peripheral blood (PB) as a sample source, has been widely used for the detection of Mtb infection worldwide (Richeldi, 2006; Wang et al., 2018b; Zhu et al., 2018). The current data support that the sensitivity of PB T-SPOT in diagnosing plTB is lower than that in diagnosing pulmonary TB (Kim et al., 2016; Hofland et al., 2017; Wang et al., 2018a). Pleural fluid (PF) can be also used to perform T-SPOT, and it seems that PF T-SPOT is better than PB T-SPOT in diagnosing plTB (Losi et al., 2007; Li et al., 2012; Zhou et al., 2015). However, this conclusion is controversial, and there was considerable heterogeneity among the studies (Aggarwal et al., 2015). Except for this, the experiment itself has a lot of uncertainty. There are even no standard protocols for PF T-SPOT and the criteria of positive and negative results of this assay are lacking.

In this study, we established the standard procedures of PF T-SPOT assay and compared it with PB T-SPOT assay in diagnosing plTB. Our data confirm that PF T-SPOT is better than PB T-SPOT or other laboratory tests in diagnosing plTB.

## Materials and Methods

### Participants

Between July 2017 and March 2018, based on symptoms and radiological abnormality (pleural effusion), 258 suspected plTB patients were consecutively recruited from Tongji hospital, Tongji medical college, Huazhong University of Science and Technology (Wuhan cohort), which is the largest tertiary hospital in central China, with 5,000 beds and a ward for patients with suspected TB. PB and PF were collected for performing PB T-SPOT and PF T-SPOT, respectively. Samples of PF, bronchoalveolar lavage, or pleural tissue were collected for performing AFS, Xpert, and Mtb culture (mycobacterial growth indicator tube and Lowenstein-Jensen media) simultaneously. Pleural tissue was also obtained for histological examination. The results of routine tests such as ADA, lactate dehydrogenase, and lymphocyte proportion were recorded. When the patients were suspected as having other diseases such as lung cancer and empyema, the relevant tests were carried out. Patients younger than 18 years of age and those undergoing TB treatment were excluded. This study was subsequently validated in another independent cohort of 253 consecutive patients who met the same inclusion criteria from Guangzhou chest hospital, the largest TB hospital in southern China with 600 beds (Guangzhou cohort). The study was approved by the Ethics Committee of Tongji Hospital, Tongji Medical College, Huazhong University of Science and Technology; and the Ethics Committee of Guangzhou chest hospital, China. All participants provided written informed consent.

### Diagnostic Criteria

The plTB patients were categorized as confirmed or probable plTB. The confirmed plTB was diagnosed if Mtb culture and/or Xpert were positive in sputum, bronchoalveolar lavage, PF, or pleural biopsy specimens. The probable plTB was diagnosed according to the following criteria: (1) although Mtb was not identified in clinical specimens, the other tests (including histological, cytological, or biochemical findings) were accordant with plTB; and (2) there was a positive response to anti-TB treatment. The non-plTB patients were diagnosed in patients who had other diagnoses (lung cancer, lymphoma, empyema, etc). The suspected plTB patients who did not fulfill the above criteria were excluded.

### PB T-SPOT Assay

PB T-SPOT assay (Oxford Immunotec, Oxford, UK) was performed according to the manufacturer's instructions. Briefly, peripheral blood mononuclear cells (PBMCs) were separated from PB using Ficoll-Hypaque density centrifugation. Then, PBMCs were counted by hemocytometer. PBMCs (2.5 × 10^5^) were added to 96-well plates precoated with anti-IFN-γ antibody. Four wells were used for each patient: positive control well (phytohemagglutinin), negative control well (medium), and two Mtb-specific antigen wells [early secreted antigenic target 6 (ESAT-6) and culture filtrate protein 10 (CFP-10)]. Spot-forming cells (SFCs) were counted with an automated ELISpot reader (CTL Analyzers, Cleveland, OH, USA). The test result was positive if ESAT-6 minus negative control and/or CFP-10 minus negative control ≥6 spots. The test result was negative if both ESAT-6 minus negative control and CFP-10 minus negative control ≤5 spots. Results were considered undetermined if the spot amounts in the positive control were <20 or if spot amounts in the negative control were >10. The final ESAT-6 or CFP-10 SFCs were defined as ESAT-6 or CFP-10 SFCs minus negative control SFCs. The MAX SFCs of PB T-SPOT was defined as the larger of final ESAT-6 and CFP-10 SFCs.

### PF T-SPOT Assay

There are no recommended procedures for performing PF T-SPOT. We have established the optimal procedures of PF T-SPOT after trying different conditions. (1) Fifty milliliter of PF was collected from patients. After centrifugation, the supernatant fluid was discarded and the pellet was resuspended in 4 ml of RPMI 1640. (2) Pleural fluid mononuclear cells (PFMCs) were separated from the cell suspension using Ficoll-Hypaque density centrifugation. (3) After two washing steps, PFMCs (1 × 10^5^) were added to 96-well T-SPOT plates, as we found that 1 × 10^5^ is the optimal number of PFMCs for performing PF T-SPOT assay. (4) The subsequent procedures were the same as those described for PB T-SPOT assay. The final ESAT-6 or CFP-10 SFCs were defined as ESAT-6 or CFP-10 SFCs minus negative control SFCs. The MAX SFCs of PF T-SPOT was defined as the same criteria as PB T-SPOT. Differently, PF T-SPOT was considered positive if MAX SFCs more than 10 and results were considered undetermined only if the spot amounts in the positive control were <20.

### Analysis of the Phenotype and Frequency of Mtb-Specific CD4^+^ T Cells

The PBMCs and PFMCs were separated from PB and PF, respectively. Monoclonal antibodies against the following antigens were added to the cell suspensions: CD4, CD45RA, CD45RO, CD62L, and CD69 (BD Pharmingen, San Diego, CA, USA). Isotype controls with irrelevant specificities were included as negative controls. All of the cell suspensions were incubated for 30 min in room temperature. For the detection of Mtb-specific cytokine production, PBMCs or PFMCs were stimulated with ESAT-6 and CFP-10 in the presence of 2 μM monensin (eBioscience, San Diego, CA, USA) for 24 h. After culture, the cells were fixed and permeabilized, and stained with anti-IFN-γ and anti-TNF-α monoclonal antibodies (eBioscience, San Diego, CA, USA). After washing, the cells were analyzed by FACSCanto flow cytometer (Becton Dickinson, San Jose, CA). Data analysis was performed using FlowJo version 7.6.1 software (TreeStar, Ashland, OR, USA).

### Statistical Analysis

Data were analyzed using GraphPad Prism 6.0 (GraphPad, La Jolla, CA, USA). Differences between groups were analyzed using the Mann-Whitney *U*-test or Student's *t*-test. The chi-square test was used for comparison of categorical data. Receiver operating characteristic (ROC) analysis was performed to determine the best threshold value of the SFCs number for distinguishing plTB from non-plTB. Area under the curve (AUC), sensitivity and specificity were reported, as well as the 95% confidence intervals (CI). Statistical significance was determined as *P* < 0.05.

## Results

### Participant Characteristics

After exclusion of 40 patients (16 with indeterminate PB or PF T-SPOT, 5 refused to receive anti-TB treatment, 8 missed follow-up, 11 without final diagnosis), 218 patients (53 confirmed plTB, 74 probable plTB, and 91 non-plTB) were diagnosed in Wuhan cohort (Supplementary Figure 1). Another 210 patients (48 confirmed plTB, 64 probable plTB, and 98 non-plTB) were diagnosed in Guangzhou cohort (Supplementary Figure 2). The etiologic distribution of pleural effusion in two cohorts is presented in Supplementary Table 1. The demographic characteristics and clinical presentations of the patients are shown in Table 1.

**Table 1:** The demographic characteristics and clinical presentations of the patients in Tongji hospital and GuangZhou chest hospital.

	**Tongji hospital (training group)**				**Guangzhou chest hospital (validation group)**				***p^**#**^***
**Variable**	**Confirmed plTB**	**Probable plTB**	**Non-plTB**	p^*^	**Confirmed plTB**	**Probable plTB**	**Non-plTB**	p^*^	
	***n*** **=** **53**	***n*** **=** **74**	***n*** **=** **91**		***n*** **=** **48**	***n*** **=** **64**	***n*** **=** **98**		**0.331**
Ages (years)	46.9 ± 22.6	43.1 ± 20.9	56.2 ± 15.4	<0.001	46.3 ± 22.3	48.8 ± 19.9	55.5± 14.9	<0.001	0.372
Gender, male	36 (67.9)	49 (66.2)	53 (58.2)	0.202	34 (70.8)	45 (70.3)	61 (62.2)	0.241	0.480
Tuberculosis history	11 (20.7)	13 (17.5)	2 (2.0)	<0.001	10 (20.8)	13 (20.3)	3 (3.1)	<0.001	1.000
**SYMPTOMS**									
Chest tightness	23 (43.4)	40 (54.0)	41 (45.1)	0.583	20 (41.7)	33 (51.6)	42 (42.9)	0.579	0.629
Chest pain	36 (67.9)	30 (40.5)	26 (28.6)	0.001	34 (70.8)	27 (42.2)	30 (30.6)	0.001	0.845
Hemoptysis	0 (0)	1 (1.4)	7 (7.7)	0.01	0 (0)	1 (1.6)	9 (9.2)	0.007	0.635
Cough	41 (77.4)	56 (75.7)	58 (63.7)	0.049	38 (79.2)	50 (78.1)	67 (68.4)	0.116	0.589
Weight loss	23 (43.4)	30 (40.5)	23 (25.3)	0.014	19 (39.6)	24 (37.5)	28 (28.6)	0.146	0.839
Fever	13 (24.5)	38 (51.4)	27 (29.7)	0.118	14 (29.2)	34 (53.1)	30 (30.6)	0.086	0.841
**RADIOLOGICAL FINDINGS**									
Lung shadow	51 (96.2)	60 (81.1)	70 (76.9)	0.046	46 (95.8)	52 (81.3)	73 (74.5)	0.02	0.705
Enlargement of lymph nodes	13 (24.5)	20 (27.0)	47 (51.6)	<0.001	12 (25.0)	17 (26.6)	58 (59.2)	<0.001	0.323
Peripheral blood WBC counts (× 10^9^/L)	6.51 ± 2.90	5.96 ± 2.96	7.27 ± 4.39	0.187	6.20 ± 2.05	6.30 ± 2.85	7.06 ± 2.82	0.084	0.314
Peripheral blood lymphocyte proportion (%)	18.3 ± 8.0	18.3 ± 8.1	20.2 ± 10.7	0.229	19.2 ± 5.8	19.6 ± 7.3	18.4 ± 9.4	0.089	0.822
hsCRP (mg/L)	52.0 ± 60.7	54.8 ± 61.4	43.2 ± 60.3	0.035	50.0 ± 50.7	45.7 ± 47.7	40.8 ± 51.6	0.043	0.948
ESR (mm/H)	42.5 ± 25.5	37.9 ± 24.4	39.8 ± 37.9	0.17	38.8 ± 25.4	36.5 ± 25.8	35.5 ± 33.6	0.167	0.343
**CYTOLOGICAL INDEXES OF PLEURAL FLUID**									
RBC counts (× 10^9^/L)	2.97 (0.80–6.82)	2.88 (1.20–5.00)	2.92 (0.61–10.8)	0.864	2.94 (1.76–3.88)	2.90 (0.79–6.41)	2.30 (0.80–14.4)	0.718	0.780
Nucleated cell counts (× 10^9^/L)	0.96 (0.60–2.25)	1.32 (0.58–2.86)	0.73 (0.36–1.92)	0.139	1.22 (0.46–2.67)	1.20 (0.52–2.00)	0.80 (0.26–1.75)	0.064	0.779
Lymphocyte proportion (%)	68.8 ± 23.8	71.9 ± 21.6	47.9 ± 25.1	<0.001	68.3 ± 19.2	70.6 ± 18.4	48.6 ± 27.7	<0.001	0.770
Neutrophils proportion (%)	8.8 ± 7.6	9.4 ± 8.2	20.6 ± 21.5	0.002	8.9 ± 8.2	11.0 ± 7.6	18.8 ± 23.4	0.349	0.875
Macrophages proportion (%)	10.0 ± 6.7	11.0 ± 8.6	20.1 ± 13.9	<0.001	11.6 ± 8.7	11.6 ± 7.2	22.6 ± 19.1	<0.001	0.469
Mesothelial cells proportion (%)	0.8 ± 1.6	1.7 ± 3.9	4.7 ± 6.0	<0.001	0.7 ± 1.4	1.4 ± 2.0	3.7 ± 6.4	0.031	0.414
Eosinophils proportion (%)	0.6 ± 1.9	0.3 ± 0.8	2.6 ± 9.0	0.18	0.3 ± 1.0	0.1 ± 0.4	3.6 ± 11.3	0.007	0.517
**BIOCHEMICAL INDEXES OF PLEURAL FLUID**									
Glucose (mmol/L)	5.1 ± 2.0	6.0 ± 1.9	6.3 ± 1.9	0.011	5.3 ± 1.6	6.1 ± 1.8	6.5 ± 2.4	0.037	0.478
Total protein (g/L)	47.8 ± 8.8	48.8 ± 6.6	34.8 ± 13.0	<0.001	49.5 ± 7.2	45.4 ± 9.9	38.6 ± 12.5	<0.001	0.711
Albumin (g/L)	26.2 ± 5.9	27.1 ± 4.2	21.7 ± 6.6	<0.001	27.2 ± 4.6	23.6 ± 8.2	22.0 ± 8.0	0.021	0.640
LDH (U/L)	426 (237–601)	367 (223–457)	156 (92–350)	<0.001	450 (297–528)	353 (179–530)	188 (125–448)	<0.001	0.273
ADA (IU/L)	26.0 ± 15.3	34.0 ± 12.4	14.2 ± 22.4	<0.001	26.3 ± 16.5	33.3 ± 18.9	13.3 ± 16.8	<0.001	0.983
CEA (ng/ml)	0.97 (0.81–1.45)	0.97 (0.63–1.57)	1.56 (0.63–22.08)	0.024	1.03 (0.58–1.76)	1.05 (0.81–2.34)	1.64 (0.83–7.7)	0.013	0.339
AFS	14 (26.4)				11 (22.9)				0.818
Xpert	42 (79.2)				37 (77.1)				0.814
Culture	48 (90.6)				42 (87.5)				0.753
Histopathology		25 (33.8)				25 (39.1)			0.595
PB T-SPOT(+)	37 (69.8)	49 (66.2)	24 (26.4)	<0.001	34 (70.8)	43 (67.2)	26 (26.5)	<0.001	0.773
PF T-SPOT(+)	50 (94.3)	70 (94.6)	27 (29.7)	<0.001	45 (93.8)	62 (96.9)	32 (32.7)	<0.001	0.837

plTB, pleural tuberculosis; WBC, white blood cell; RBC, red blood cell; hsCRP, hypersensitive C reactive protein; ESR, erythrocyte sedimentation rate; ADA, adenosine deaminase activity; LDH, lactate dehydrogenase; CEA, carcinoembryonic antigen; AFS, acid-fast stain; PB, peripheral blood; PF, pleural fluid.

* Comparisons were performed between plTB group (including confirmed plTB and probable plTB) and non-plTB group using chi-square test and Mann–Whitney U test.

# *Comparisons were performed between the Wuhan and Guangzhou cohorts using chi-square test and Mann–Whitney U test. Data are presented as means ± SD, medians (25th−75th centiles) or number (percentage)*.

### Determination of the Optimal Number of PFMCs for Performing PF T-Spot Assay

**Figure 1:**
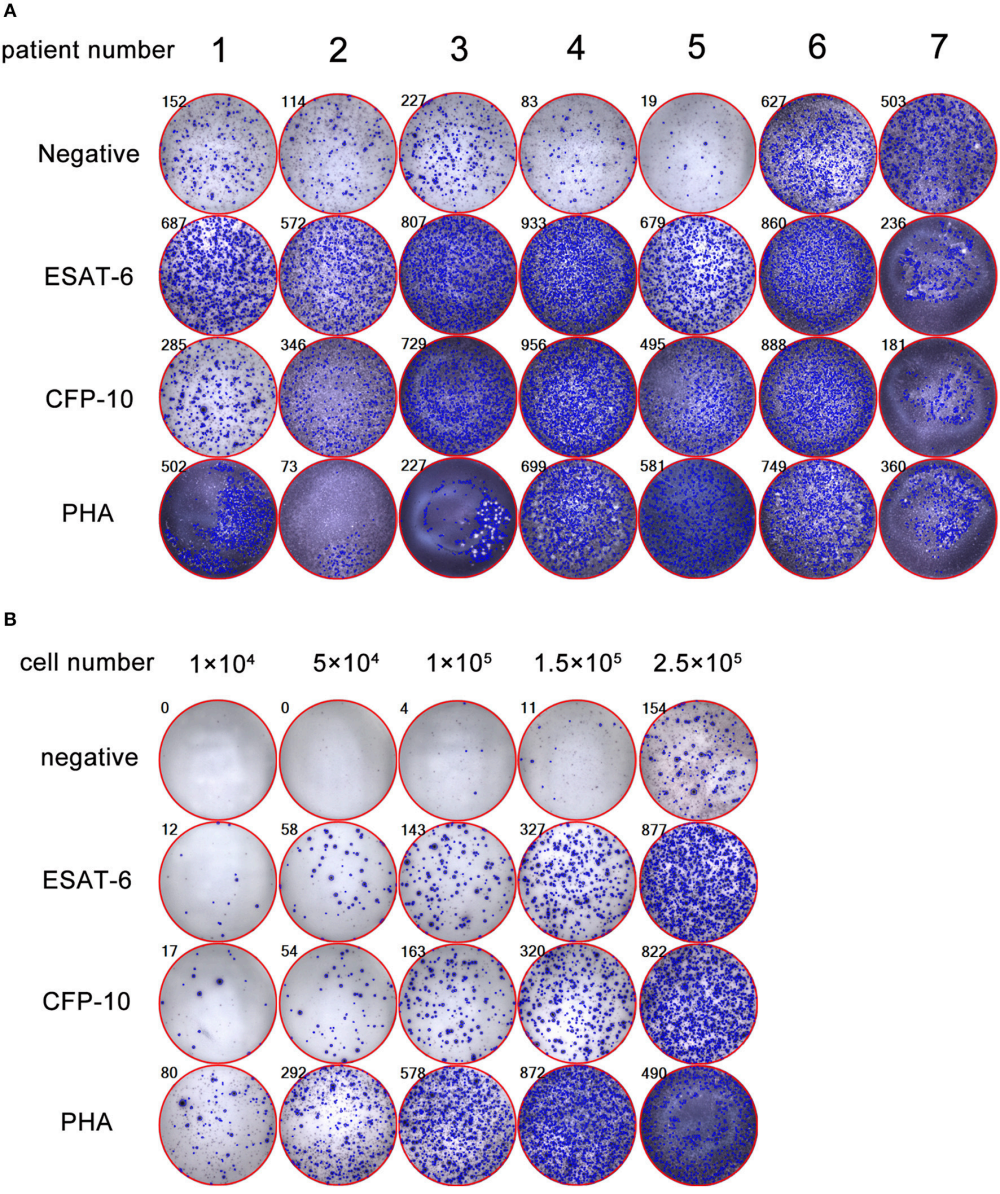
Determination of the optimal number of PFMCs for performing PF T-SPOT. **(A)** Representative pictures showing inaccurate PF T-SPOT results If 2.5 × 10^5^ PFMCs are added to T-SPOT well (patient 1: PHA SFCs are inaccurate; patient 2 to 5: Mtb-specific antigens and PHA SFCs are inaccurate; patient 6 and 7: negative well, Mtb-specific antigens, and PHA SFCs are inaccurate). **(B)** The PF T-SPOT results under different number of PFMCs. Different number of PFMCs (1 × 10^4^, 5 × 10^4^, 1 × 10^5^, 1.5 × 10^5^, 2.5 × 10^5^) of one representative patient was added to T-SPOT well. The number in the upper left corner of each graph indicates the number of SFC in each well of PF T-SPOT assay. ESAT-6, early secreted antigenic target 6; CFP-10, culture filtrate protein 10; PHA, phytohaemagglutinin.

Due to there are no standard procedures for performing PF T-SPOT, we had to establish our own protocol and found that the key point affected this assay is the number of PFMCs added to each well. We demonstrated that 1 × 10^5^ is the optimal number of PFMCs for performing PF T-SPOT, which is the biggest difference between PF T-SPOT and PB T-SPOT. The following representative pictures show that if 2.5 × 10^5^ PFMCs are added to T-SPOT well, then it is impossible for ELISpot reader to count spots accurately because too many spots are crowded in one well: (1) PHA SFCs cannot be counted (patient 1); (2) both Mtb-specific antigens and PHA SFCs cannot be counted (patient 2 to 5); and (3) negative, Mtb-specific antigens, and PHA SFCs cannot be counted (patient 6 and 7) (Figure 1A). We have also tried different numbers of PFMCs added to each well. As shown in Figure 1B, we found that 1 × 10^5^ is the optimal number of PFMCs added to each well (5 × 10^4^, too low SFCs; 1.5 × 10^5^, too many SFCs).

### Using PB T-Spot and PF T-Spot in Diagnosing plTB

**Figure 2:**
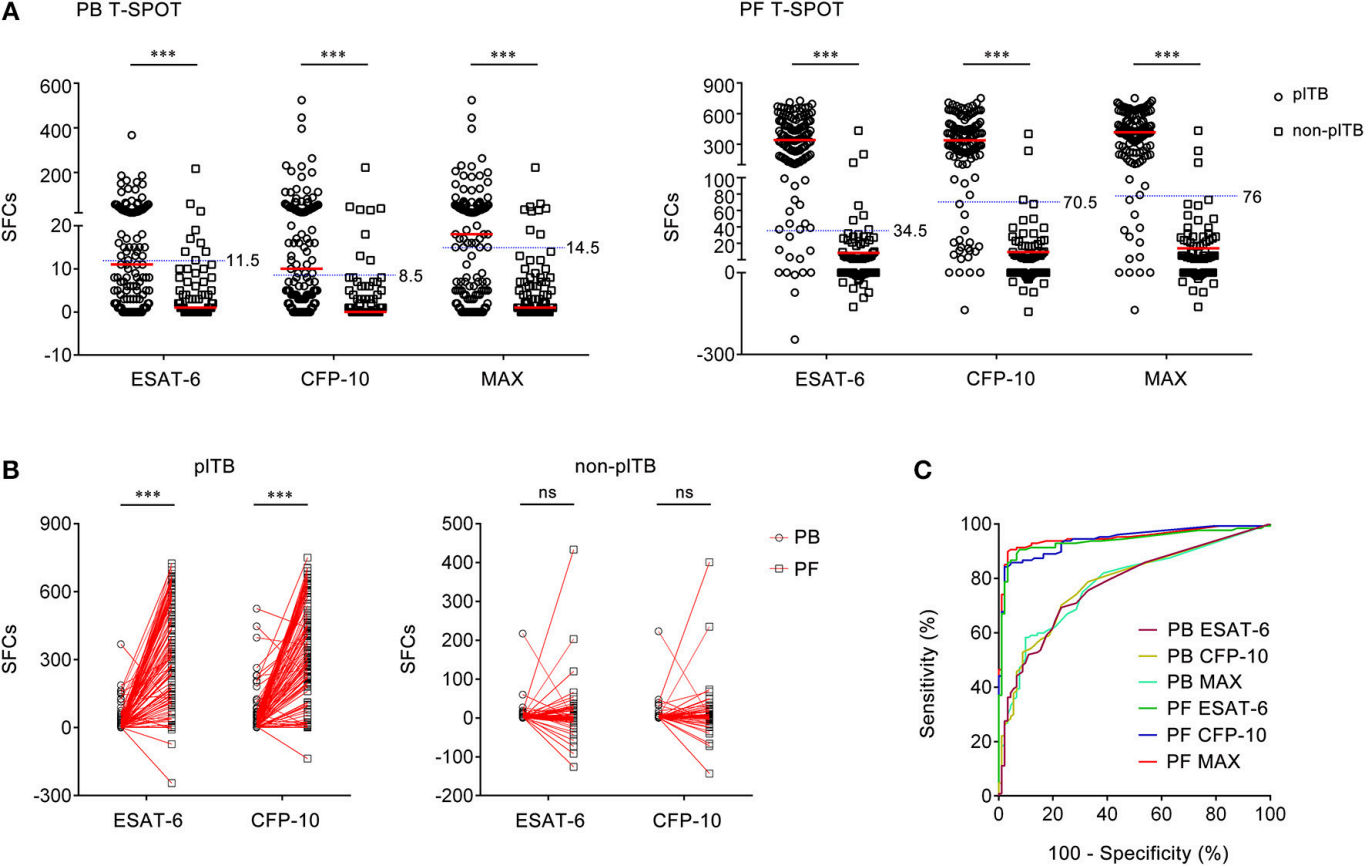
The results of PB T-SPOT and PF T-SPOT in Wuhan cohort. **(A)** Scatter plots showing ESAT-6 SFCs, CFP-10 SFCs, and MAX SFCs in PB T-SPOT and PF T-SPOT in plTB (*n* = 127) and non-plTB (*n* = 91) patients. Horizontal lines indicate the median. ^***^P < 0.001 (Mann–Whitney *U*-test). Blue dotted lines indicate the cutoff values in distinguishing these two groups. **(B)** Line graphs showing the results of ESAT-6 and CFP-10 SFCs for each patient in PB and PF T-SPOT. One line represents one patient. ^***^P < 0.001; ns, no significance (Student's *t*-test). **(C)** ROC analysis showing the performance of ESAT-6 SFCs, CFP-10 SFCs, and Max SFCs in PB and PF T-SPOT in diagnosing plTB. PB, peripheral blood; PF, pleural fluid; T-SPOT, T-SPOT.TB; plTB, pleural tuberculosis; ESAT-6, early secreted antigenic target 6; CFP-10, culture filtrate protein 10; SFCs, spot-forming cells; MAX, the larger of ESAT-6 and CFP-10.

As some of the antigen SFCs are less than negative control SFCs in PF T-SPOT, the final ESAT-6 or CFP-10 SFCs are occasionally below zero, especially in non-plTB patients. However, the final ESAT-6, CFP-10, and MAX SFCs of both PB T-SPOT and PF T-SPOT in plTB patients were significantly higher than those in non-plTB patients (Figure 2A). Furthermore, both ESAT-6 and CFP-10 SFCs were significantly increased in PF T-SPOT when compared to PB T-SPOT in plTB patients, while no statistical difference was observed in these values in non-plTB patients (Figure 2B).

Although ESAT-6, CFP-10, and MAX SFCs in both PB T-SPOT and PF T-SPOT had significant difference between plTB and non-plTB patients, ROC analysis showed that the performance of ESAT-6, CFP-10, and MAX in PB T-SPOT was unsatisfactory in distinguishing these two conditions (Figure 2C). The AUC of ESAT-6 was 0.776, with a sensitivity of 48.82% and a specificity of 90.11%. The AUC of CFP-10 was 0.783, with a sensitivity of 52.76% and a specificity of 91.21%. In addition, according to the manufacturer's cutoff value, the diagnostic sensitivity and specificity of PB T-SPOT were also poor (Table 2). As expected, the performance of PF T-SPOT was obviously improved. MAX in PF T-SPOT showed the best diagnostic efficiency. The AUC of MAX was 0.950, with a sensitivity of 89.76% and a specificity of 96.70% when a cutoff value of 76 was used (Figure 2C, Table 2). These data suggest that the performance of PF T-SPOT assay is obviously better than PB T-SPOT assay in diagnosing plTB.

**Table 2:** Diagnostic performance of different indexes in the diagnosis of plTB in Wuhan cohort.

**Variable**	**Cutoff value**	**AUC (95% CI)**	**Sensitivity (%)**	**Specificity (%)**	**PPV (%)**	**NPV (%)**
PB T-SPOT	6^*^	NA	67.72	71.43	76.79	61.32
PB ESAT-6 SFCs	11.50	0.776 (0.714–0.838)	48.82	90.11	87.32	55.78
PB CFP-10 SFCs	8.50	0.783 (0.722–0.844)	52.76	91.21	89.33	58.04
PB MAX SFCs	14.50	0.779 (0.718–0.841)	58.27	90.11	89.16	60.74
PF ESAT-6 SFCs	34.50	0.937 (0.902–0.972)	90.55	92.31	94.26	87.50
PF CFP-10 SFCs	70.50	0.942 (0.911–0.973)	84.25	96.70	97.27	81.48
PF MAX SFCs	76.00	0.950 (0.919–0.980)	89.76	96.70	97.44	87.13

plTB, pleural tuberculosis; AUC, area under the curve; CI, confidence interval; ESAT-6, early secretory antigenic target 6; CFP-10, culture filtrate protein 10; PB, peripheral blood; PF, pleural fluid; SFCs, spot-forming cells; PPV, positive predictive value; NPV, negative predictive value; NA, not applicable;

* *manufacturer's cutoff value, ESAT-6 or CFP-10 ≥ 6*.

### The Phenotype and Frequency of Mtb-Specific Cd4^+^ T Cells in PB and PF

**Figure 3:**
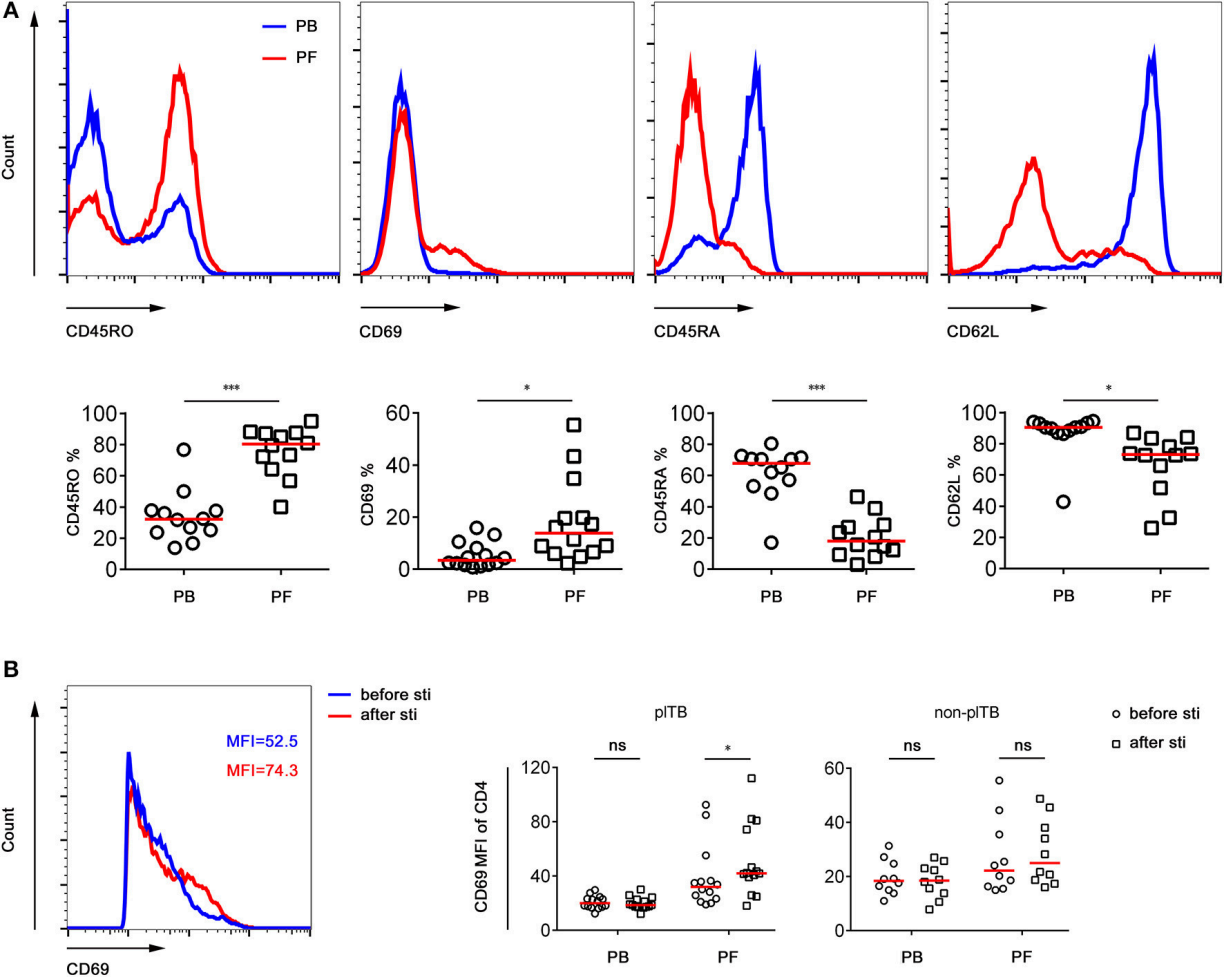
The phenotype of CD4^+^ T cells in PB and PF. **(A)** PB and PF were collected from plTB patients. PBMCs and PFMCs were separated for analysis of the phenotypic characteristics of CD4^+^ T cells. Representative of FACS histogram showing the expression of CD45RO, CD69, CD45RA, and CD62L on CD4^+^ T cells. The percentages of CD45RO^+^, CD69^+^, CD45RA^+^, CD62L^+^ cells in CD4^+^ T cells in PB and PF are shown. Horizontal lines indicate the median. ^*^P < 0.05; ^***^P < 0.001 (Mann–Whitney *U*-test). **(B)** PBMCs and PFMCs separated from plTB and non-plTB patients were stimulated with Mtb-specific antigen (ESAT-6 and CFP-10 complex) for 24 h. The expression of CD69 on CD4^+^ T cells before and after stimulation was analyzed. Scatter plots showing the MFI of CD69 on CD4^+^ T cells in PB and PF from plTB and non-plTB patients. ^*^P < 0.05; ns, no significance (Mann–Whitney *U*-test). PB, peripheral blood; PF, pleural fluid; plTB, pleural tuberculosis; sti, stimulation; MFI, mean fluorescence intensity.

The phenotype and frequency of Mtb-specific lymphocytes collected from PB and PF were further determined. We observed that the expression of activation markers CD45RO and CD69 on CD4^+^ T cells in PF was significantly higher than that in PB. In contrast, the expression of naïve marker CD45RA and lymphoid homing marker CD62L on CD4^+^ T cells in PF was obviously lower than that in PB (Figure 3A). These data demonstrate that CD4^+^ T cells in PF are more activated than those in PB in plTB patients.

The expression of CD69 on CD4^+^ T cells in PF from plTB patients was significantly increased after Mtb-specific antigen stimulation. However, the expression of CD69 on CD4^+^ T cells had no statistical difference between before and after stimulation in both PB and PF from non-plTB patients (Figure 3B).

**Figure 4:**
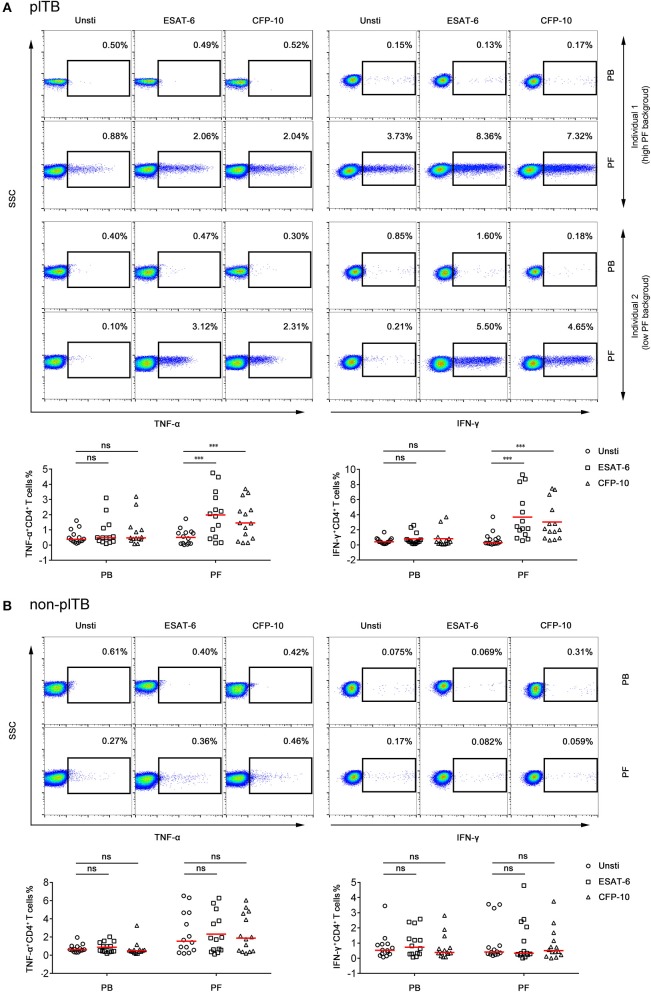
The frequency of Mtb-specific CD4^+^ T cells in PB and PF. PBMCs and PFMCs separated from plTB and non-plTB patients were stimulated with ESAT-6 and CFP-10 for 24 h. **(A)** Representative FACS plots showing the expression of TNF-α and IFN-γ in CD4^+^ cells in plTB patients. Individual 1 represents a high background patient and individual 2 represents a low background patient. The percentages of TNF-α^+^ and IFN-γ^+^ cells in CD4^+^ cells are shown. Horizontal lines indicate the median. **(B)** Representative FACS plots showing the expression of TNF-α and IFN-γ in CD4^+^ cells in non-plTB patients. The percentages of TNF-α^+^ and IFN-γ^+^ cells in CD4^+^ cells are shown. Horizontal lines indicate the median. ^***^P < 0.001; ns, no significance (Mann–Whitney *U*-test). PB, peripheral blood; PF, pleural fluid; plTB, pleural tuberculosis; Unsti, unstimulation; ESAT-6, early secreted antigenic target 6; CFP-10, culture filtrate protein 10.

The percentages of IFN-γ^+^ or TNF-α^+^ CD4^+^ T cells in PF from plTB patients were significantly increased after ESAT-6 or CFP-10 stimulation. However, the production of these cytokines in CD4^+^ T cells in PB from plTB patients had no statistical difference after stimulation (Figure 4A). The production of cytokines in CD4^+^ T cells in both PB and PF from non-plTB patients also had no significant change after stimulation (Figure 4B). These data suggest that the frequency of Mtb-specific lymphocytes in PF is higher than that in PB, which might result in improved performance of PF T-SPOT in diagnosing plTB.

### Comparison of Diagnostic Performance Between PF T-Spot and Other Tests

As shown in Table 3, AFS had the lowest sensitivity (11.02%) as a single test for diagnosing plTB. The sensitivities of Xpert and Mtb culture were similar and around 35%. The sensitivity of combination of Xpert and Mtb culture was still low (41.73%). The percentage of lymphocyte and ADA level in PF also showed poor diagnostic performance because of either low sensitivity or low specificity. Interestingly, PF T-SPOT assay showed a high sensitivity (89.76%) as a single test for diagnosing plTB, and the specificity (96.70%) of this test was also satisfactory.

**Table 3:** The performance of PF T-SPOT assay and routine tests in the diagnosis of plTB.

	**Sensitivity (%)**	**Specificity (%)**
AFS	11.02	100
Xpert	33.07	100
Mtb culture	37.80	100
Xpert and Mtb culture	41.73	100
PF T-SPOT	89.76	96.70
L% in PF (>50%)	78.74	57.14
ADA level in PF (>30 IU/L)	59.06	91.21

*PF, pleural fluid; plTB, pleural tuberculosis; AFS, acid-fast stain; L%, the percentage of lymphocyte*.

### Validation of PF T-Spot Assay in Another Center

**Figure 5:**
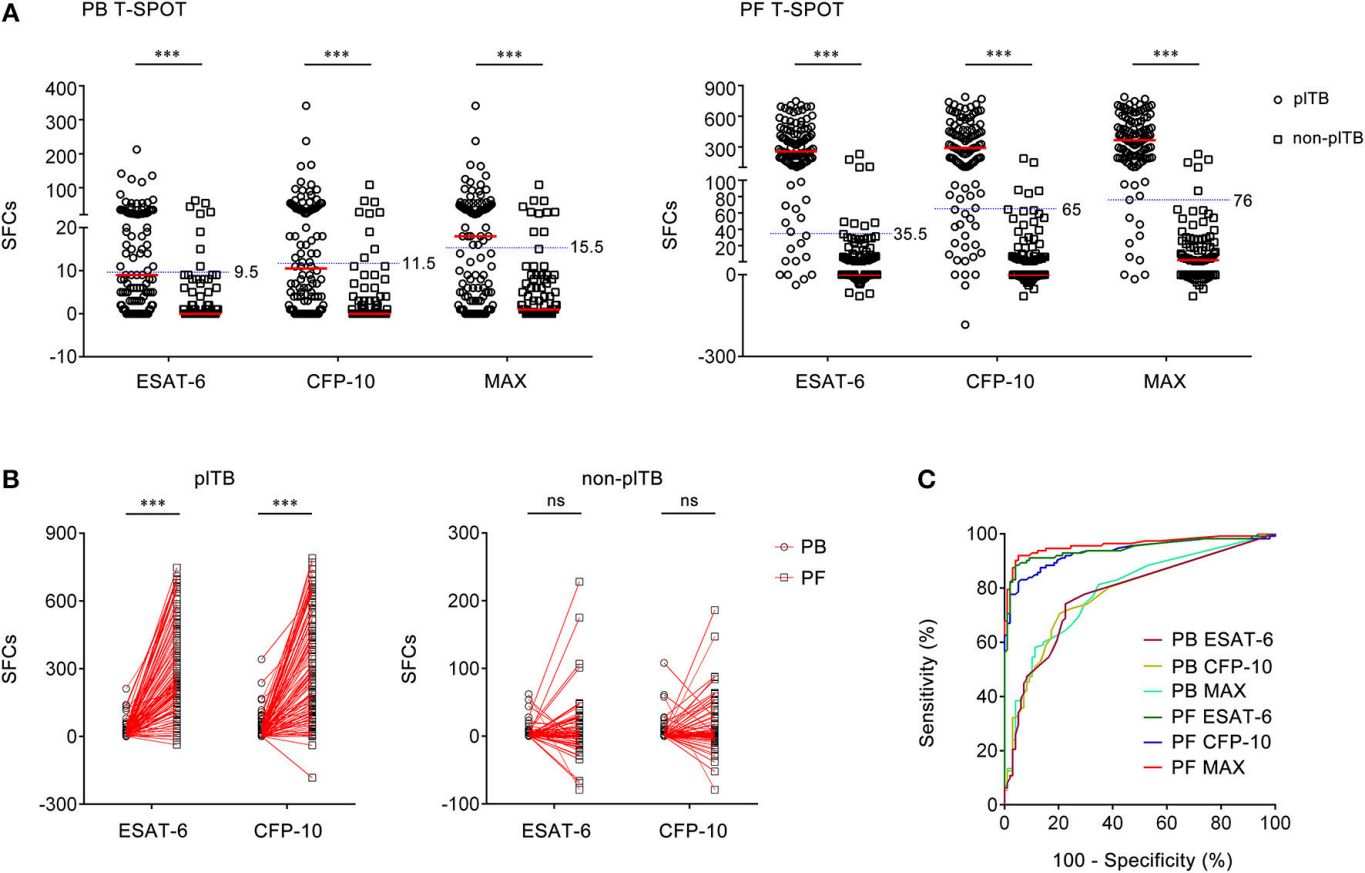
The results of PB T-SPOT and PF T-SPOT in Guangzhou cohort. **(A)** Scatter plots showing ESAT-6 SFCs, CFP-10 SFCs, and MAX SFCs in PB T-SPOT and PF T-SPOT in plTB (*n* = 112) and non-plTB (*n* = 98) patients. Horizontal lines indicate the median. ^***^P < 0.001 (Mann–Whitney *U*-test). Blue dotted lines indicate the cutoff values in distinguishing these two groups. **(B)** Line graphs showing the results of ESAT-6 and CFP-10 SFCs for each patient in PB and PF T-SPOT. One line represents one patient. ^***^P < 0.001; ns, no significance (Student's *t*-test). **(C)** ROC analysis showing the performance of ESAT-6 SFCs, CFP-10 SFCs, and Max SFCs in PB and PF T-SPOT in diagnosing plTB. PB, peripheral blood; PF, pleural fluid; T-SPOT, T-SPOT.TB; plTB, pleural tuberculosis; ESAT-6, early secreted antigenic target 6; CFP-10, culture filtrate protein 10; SFCs, spot-forming cells; MAX, the larger of ESAT-6 and CFP-10.

A second group of independent patients were recruited as validation cohort in Guangzhou chest hospital. Similarly, although the results of both PB T-SPOT and PF T-SPOT in plTB patients were significantly higher than those in non-plTB patients, ROC analysis showed that the performance of ESAT-6, CFP-10, and MAX in PB T-SPOT had limited value in diagnosing plTB (Figures 5A,C). However, the results of PF T-SPOT were significantly increased compared with PB T-SPOT in plTB patients (Figure 5B). When the cutoff value of MAX SFCs was set at 76, the AUC of PF T-SPOT was 0.961, with a sensitivity of 91.07% and a specificity of 94.90% in distinguishing plTB from non-plTB (Figure 5C and Table 4). These data confirm that PF T-SPOT assay has a prominent role in diagnosing plTB.

**Table 4:** Diagnostic performance of different indicators in the diagnosis of plTB in Guangzhou cohort.

**Variable**	**Cutoff value**	**AUC (95% CI)**	**Sensitivity (%)**	**Spesitivity (%)**	**PPV (%)**	**NPV (%)**
PB T-SPOT	6^*^	NA	68.75	72.45	74.04	66.98
PB ESAT-6 SFCs	9.5	0.779 (0.715–0.842)	47.32	91.84	86.89	60.40
PB CFP-10 SFCs	11.5	0.780 (0.717–0.843)	47.32	90.82	85.48	60.14
PB MAX SFCs	15.5	0.791 (0.730–0.852)	54.46	89.80	85.92	63.31
PF ESAT-6 SFCs	35.5	0.944 (0.911–0.978)	90.18	91.84	92.66	89.11
PF CFP-10 SFCs	65	0.935 (0.900–0.969)	82.14	94.90	94.85	82.30
PF MAX SFCs	76	0.961 (0.934–0.988)	91.07	94.90	95.33	90.29

plTB, pleural tuberculosis; AUC, area under the curve; CI, confidence interval; ESAT-6, early secretory antigenic target 6; CFP-10, culture filtrate protein 10; PB, peripheral blood; PF, pleural fluid; SFCs, spot-forming cells; PPV, positive predictive value; NPV, negative predictive value; NA, not applicable;

* *manufacturer's cutoff value, ESAT-6 or CFP-10 ≥ 6*.

## Discussion

Pleural tuberculosis is the main cause of pleural effusion in most countries. However, the diagnosis of plTB is still a challenge (Solovic et al., 2013). The pathogenic tests (AFS, Mtb culture, and Xpert) or other laboratory markers (lymphocyte % and ADA level in PF) have limited value in diagnosing the disease. In the present study, we confirm that the diagnostic efficiency of PF T-SPOT assay is obviously better than PB T-SPOT assay or other laboratory tests, which suggests that PF T-SPOT assay has a great potential in the diagnosis of plTB.

It has become more and more clear that the performance of PB T-SPOT in diagnosing plTB is unsatisfactory. Although some studies have found a high sensitivity (>90%) but a moderate specificity (≈70%) of this test in diagnosing plTB (Liu et al., 2013; He et al., 2015), others studies have shown that both sensitivity and specificity are moderate (≈70%) (Dheda et al., 2009; Zhang et al., 2014). A recent meta-analysis has revealed that the pooled sensitivity and specificity of PB T-SPOT are 77 and 71%, respectively (Aggarwal et al., 2015). In accordance with this, our data showed that the optimal sensitivity and specificity of PB T-SPOT were approximately 50 and 90%. Besides, our previous study has also showed that the optimal sensitivity and specificity of PB T-SPOT in diagnosing plTB are 60 and 70% (Wang et al., 2018a). All of these data indicate that PB T-SPOT assay has only moderate sensitivity or specificity in diagnosing plTB.

The performance of PF T-SPOT in diagnosing plTB is inconsistent. Several studies have shown that the diagnostic accuracy of PF T-SPOT is better than PB T-SPOT (Lee et al., 2009; Kang et al., 2012; Liu et al., 2013; Liao et al., 2014; Adilistya et al., 2016; Kim et al., 2016). A meta-analysis has showed that the pooled sensitivity and specificity of PF T-SPOT are 93 and 90% (Li et al., 2015), while another one has reported that the pooled sensitivity and specificity of PF IFN-γ release assay are only 72 and 78% and that there is considerable heterogeneity among the studies (Aggarwal et al., 2015). We found that the most important reason caused the discrepancy is that some of the data come from QuantiFERON (another World Health Organization-recommended IFN-γ release assay). But actually, the sensitivity of PF QuantiFERON is obviously lowed than that of PF T-SPOT. The following two reasons can be considered: (1) the variable dilutions of PF lymphocytes may result in more indeterminate or false-negative results of QuantiFERON; and (2) a high background of IFN-γ production in unstimulated tube may contribute to indeterminate or false-negative results. However, these influences can be eliminated by purifying PFMCs in PF T-SPOT assay.

Furthermore, the following reasons can be used to explain why the performance of PF T-SPOT is varied among the studies. First, there are no standard instructions for performing PF T-SPOT. Thus, different studies could use different procedures. For instance, one study may add 1 × 10^5^ PFMCs to T-SPOT well, while another may add 2 × 10^5^ PFMCs to it, which will cause apparent discrepancies in PF T-SPOT results. Second, as it is difficult to collect enough PF in some patients, in a previous study, only 1 × 10^3^ PFMCs were added to T-SPOT well (Lee et al., 2009). This might decrease the sensitivity of PF T-SPOT in diagnosing plTB. Third, there is no criterion for understanding PF T-SPOT results. Different groups could have different PF T-SPOT results, even for the same number of spot (Kang et al., 2012; Keng et al., 2013; Liu et al., 2013; Liao et al., 2014; Kim et al., 2016).

Two important issues should be mentioned. One is that PF T-SPOT results are considered indeterminate only if the spot amounts in the positive control were <20. High value in the negative control is regarded as available PF T-SPOT results in our study, which is one of the differences between PF T-SPOT and PB T-SPOT. Another is that some non-plTB patients also had positive PF T-SPOT results. This may be caused by the entrance of Mtb-specific cells from blood into pleural cavity due to inflammation or hemorrhage. However, although some non-plTB patients may have positive PF T-SPOT, we noticed that the results of both ESAT-6 and CFP-10 SFCs in these patients were low. Thus, we also have compared the ratio of PF SFCs to PB SFCs between plTB and non-plTB patients. As expected, we found that the ratio of PF SFCs to PB SFCs in plTB patients is significantly higher than that in non-plTB patients (Supplementary Figure 3). Moreover, after comparing the performance of MAX SFCs and sum of ESAT-6 and CFP-10 SFCs, we found that MAX SFCs is better than sum of ESAT-6 and CFP-10 SFCs in diagnosing plTB. Thus, we used MAX SFCs instead of sum of ESAT-6 and CFP-10 SFCs in this study.

There are several limitations to this study. First, although this is a multi-center study, the sample size is actually small in each center. Second, less than half of the plTB patients were diagnosed according to positive Mtb culture or Xpert results, which may be caused by the pauci-bacillary nature of the disease. Another reason may be that pleural effusion instead of pleural tissue is used for performing the above tests occasionally due to the difficulty in collecting tissue. However, the positive rate of Mtb culture or Xpert could be decreased in this condition.

In general, this study demonstrates that the performance of PF T-SPOT assay is obviously better than PB T-SPOT assay and other tests, which suggests that PF T-SPOT assay has been of great value in diagnosing plTB.

## Author Contributions

YL, YT, JY, and QL: performed experiments and analyzed data. YL, HH, LM, WL, FW, and ZS: developed the concept, designed the study, analyzed data, and wrote the paper. All the authors commented on the paper.

### Conflict of Interest Statement

The authors declare that the research was conducted in the absence of any commercial or financial relationships that could be construed as a potential conflict of interest.

## Supplementary Material

Supplementary Figure 1 Flow diagram summarizing patient recruitment, exclusion criteria and the patient groups in Wuhan cohort.

Supplementary Figure 2 Flow diagram summarizing patient recruitment, exclusion criteria and the patient groups in Gugangzhou cohort.

Supplementary Figure 3 The ratio of PF SFCs to PB SFCs in Wuhan and Guangzhou cohort. **(A)** Scatter plots showing the ratios of PF SFCs to PB SFCs between plTB and non-plTB patients in Wuhan cohort and **(B)** Guangzhou cohort. Horizontal lines indicate the median. ^***^*P* < 0.001 (Mann–Whitney *U*-test). PB, peripheral blood; PF, pleural fluid; plTB, pleural tuberculosis; ESAT-6, early secreted antigenic target 6; CFP-10, culture filtrate protein 10.

Supplementary Table 1 The etiologic distribution of pleural effusion in two cohorts.
