# Supplementary figures and images for: The Performance of Pleural Fluid T-SPOT.TB Assay for Diagnosing Tuberculous Pleurisy in China: A Two-Center Prospective Cohort Study
Source: Front Cell Infect Microbiol. 2019 Jan 30;9:10. doi: 10.3389/fcimb.2019.00010 (PMC6363671; doi:10.3389/fcimb.2019.00010)

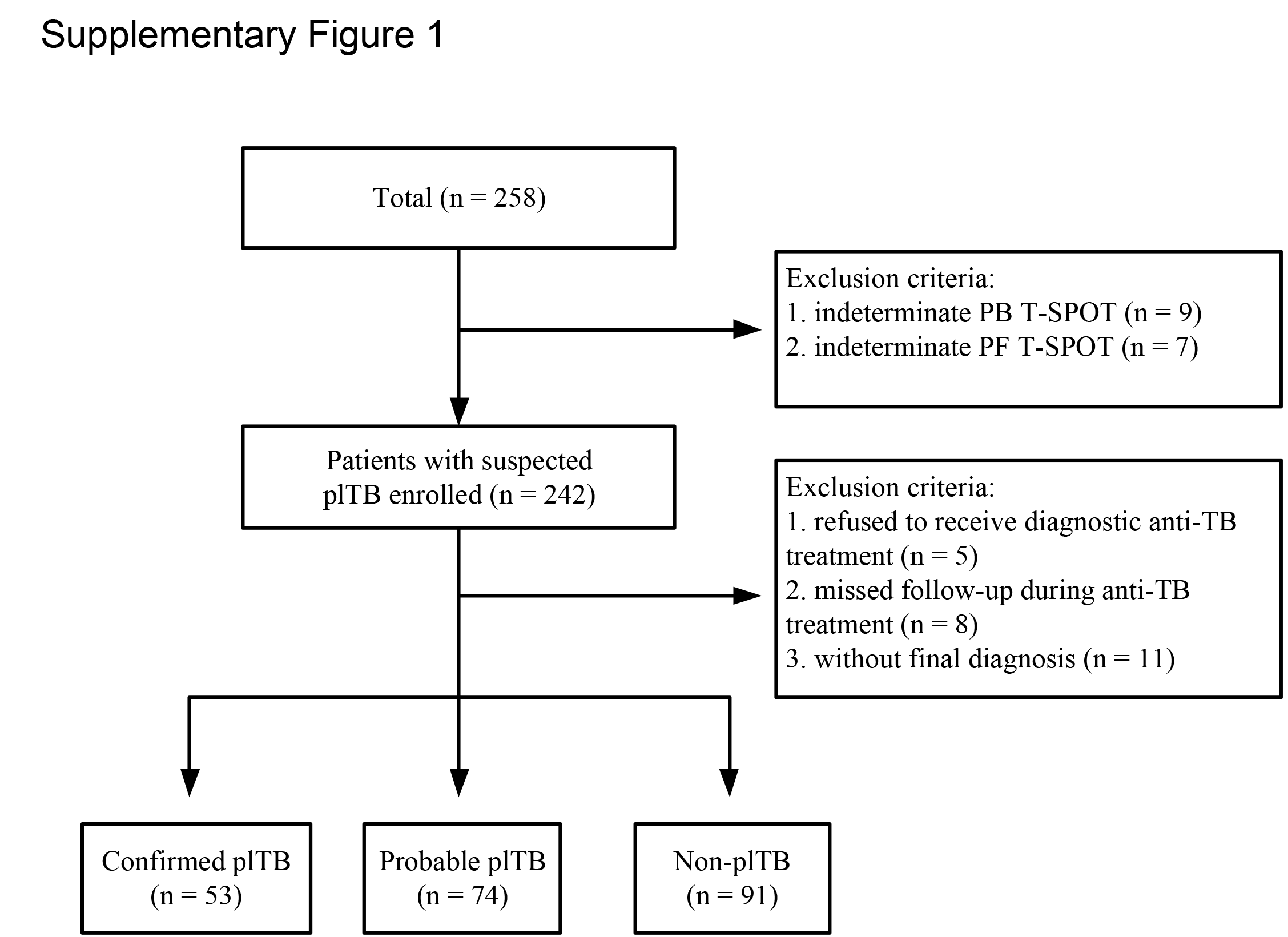

Supplement: Supplementary Figure 1 — Flow diagram summarizing patient recruitment, exclusion criteria and the patient groups in Wuhan cohort. [file Image_1.TIF]

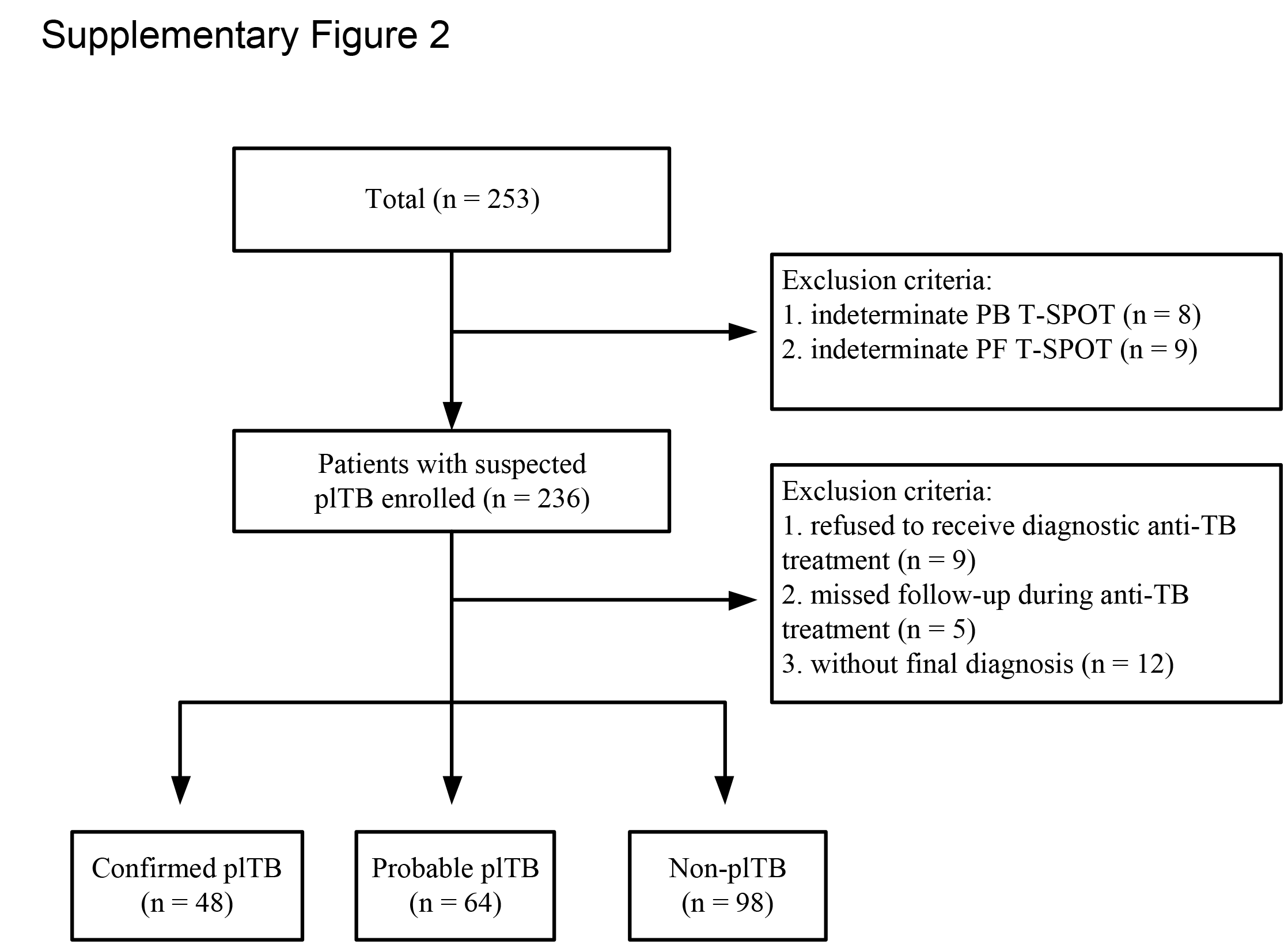

Supplement: Supplementary Figure 2 — Flow diagram summarizing patient recruitment, exclusion criteria and the patient groups in Gugangzhou cohort. [file Image_2.TIF]

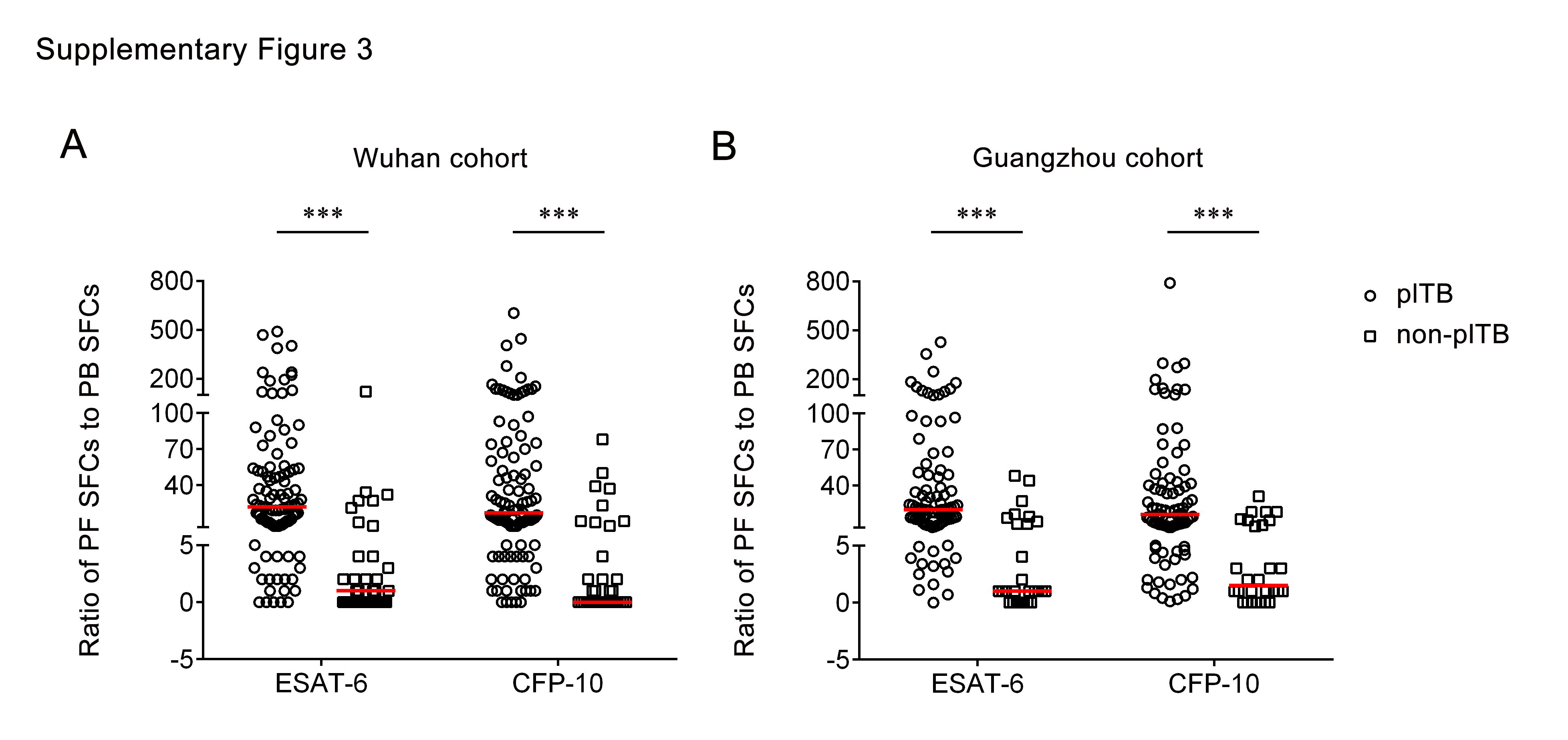

Supplement: Supplementary Figure 3 — The ratio of PF SFCs to PB SFCs in Wuhan and Guangzhou cohort. (A) Scatter plots showing the ratios of PF SFCs to PB SFCs between plTB and non-plTB patients in Wuhan cohort and (B) Guangzhou cohort. Horizontal lines indicate the median. ***P < 0.001 (Mann–Whitney U-test). PB, peripheral blood; PF, pleural fluid; plTB, pleural tuberculosis; ESAT-6, early secreted antigenic target 6; CFP-10, culture filtrate protein 10. [file Image_3.TIF]
